# Methylation patterns at fledging predict delayed dispersal in a cooperatively breeding bird

**DOI:** 10.1371/journal.pone.0252227

**Published:** 2021-06-04

**Authors:** Andrea L. Liebl, Jeff S. Wesner, Andrew F. Russell, Aaron W. Schrey

**Affiliations:** 1 Department of Biology, University of South Dakota, Vermillion, South Dakota, United States of America; 2 Centre for Ecology and Conservation, University of Exeter, Penryn, Cornwall, United Kingdom; 3 Department of Biology, Georgia Southern University, Armstrong, Georgia, United States of America; University of Tulsa, UNITED STATES

## Abstract

Individuals may delay dispersing from their natal habitat, even after maturation to adulthood. Such delays can have broad consequences from determining population structure to allowing an individual to gain indirect fitness by helping parents rear future offspring. Dispersal in species that use delayed dispersal is largely thought to be opportunistic; however, how individuals, particularly inexperienced juveniles, assess their environments to determine the appropriate time to disperse is unknown. One relatively unexplored possibility is that dispersal decisions are the result of epigenetic mechanisms interacting between a genome and environment during development to generate variable dispersive phenotypes. Here, we tested this using epiRADseq to compare genome-wide levels of DNA methylation of blood in cooperatively breeding chestnut-crowned babblers (*Pomatostomus ruficeps*). We measured dispersive and philopatric individuals at hatching, before fledging, and at 1 year (following when first year dispersal decisions would be made). We found that individuals that dispersed in their first year had a reduced proportion of methylated loci than philopatric individuals before fledging, but not at hatching or as adults. Further, individuals that dispersed in the first year had a greater number of loci change methylation state (i.e. gain or lose) between hatching and fledging. The existence and timing of these changes indicate some influence of development on epigenetic changes that may influence dispersal behavior. However, further work needs to be done to address exactly how developmental environments may be associated with dispersal decisions and which loci in particular are manipulated to generate such changes.

## Introduction

How animals move and disperse has broad implications for both the individual (e.g. habitat) and the population (e.g. population structure). Dispersal itself is likely influenced by a myriad of factors relating to individual internal and external environment, including the genome [1–4]. Indeed, research suggests a relationship between dispersal and particular gene types such as lipid metabolism and antigen defense [5]. In some species, family groups are dependent on sexually mature individuals delaying dispersal from their natal habitat (herein referred to as delayed dispersal) to remain with breeding parents and delay their own breeding. Such dispersal decisions have additional implications for the ecology and evolution of the species as a whole- from population dynamics and structure to the evolution of cooperative breeding [6]. Individuals may delay or forego dispersal when the benefits of remaining philopatric outweigh those of dispersing; these benefits might include the indirect fitness gained by helping rear relatives, enhanced foraging efficiency, reduced predation risk, the probability of inheriting a high-quality breeding position, or even parental practice [7]. However, even within a species with delayed dispersal, individuals can vary in how long they postpone independence, and the timing of dispersal has been thought to be largely driven by post-developmental opportunism, in that offspring remain with their natal group until a suitable breeding opportunity arises [8–14]. Therefore, environmental cues likely play a large role in shaping how and when individuals disperse from their natal environments. However, how environmental cues are assessed and synthesized to determine the appropriate timing of dispersal, particularly by inexperienced juveniles, is largely unknown.

Research across taxa suggests that epigenetic processes, such as DNA methylation, are one way environments interact with the genome to generate variable phenotypes [15–22]. Epigenetic changes can strengthen, weaken, or entirely silence gene expression in response to some environmental stimuli, imparting consequences for how a genome is expressed into a phenome. Although some epigenetic changes occur rapidly in response to changing environments (including social environment [19, 23]), others are stable through time, allowing exposure to a single environment to induce an epigenetic change that can dictate a specific phenotypic trajectory throughout life [24]. In fact, differences in developmental environment can induce variable social and parental behavior via epigenetic mechanisms [25–27]. Even the reproductive castes that drive eusociality (an extreme version of cooperative behavior) in honeybees are regulated epigenetically via alterations to patterns of DNA methylation by the consumption of royal jelly early in life [18]. However, more research is needed on the impact of developmental environment on differential methylation and how that relates to variation in dispersal behavior and its consequences.

Here, we test whether or not molecular epigenetic interactions between the environment and the genome influence dispersal decisions in a cooperatively breeding vertebrate. Delayed dispersal is particularly important in cooperatively breeding species [6, 28] as 96% of the bird species in which offspring remain with their parents into adulthood also cooperatively breed [29]. Individuals of cooperatively breeding species have three options on reaching adulthood: disperse to breed; disperse without breeding; or, delaying dispersal, remaining philopatric and helping [7]. If individuals that remain gain indirect fitness benefits by helping their parents raise future offspring, and this fitness benefit outweighs the fitness that may be gained by dispersing, cooperative breeding should evolve [30, 31]. Delaying dispersal to help is most likely to evolve when available high quality habitats are rare [7] and/or when environmental predictability is low [6], making breeding without help unlikely to be successful.

Here we use the chestnut-crowned babbler (*Pomatostomus ruficeps*), a bird endemic to arid and semi-arid Australia, where breeding relies on seasonal rainfall. This species does not rely on limited resources (e.g. nesting holes) for breeding or survival, are slow to colonize vacant habitat, and are not observed alone, outside social groups [32]. Although among individual variation exists in timing of dispersal in this species [33], there are no obvious socio-ecological predictors [32], and some individuals never disperse. Breeding groups consist of a single breeding female, between one and three breeding males, and between two and 15 (mean = 6) non-breeding adults that care for offspring by provisioning [34]. Non-breeding helpers are often offspring that have delayed dispersal [35], highlighting the importance of delayed dispersal in cooperative behavior for this species. As a mechanism that interacts between variable environments and the genome, we expected that differences in genome-wide levels of methylation would predict dispersal behavior in chestnut-crowned babblers. Further, we anticipated that these differences in methylation would arise during development, such that no differences in methylation between individuals that dispersed and those that remained philopatric would be evident at hatching but would be before chicks fledged the nest. Understanding how individuals make decisions on when to disperse has implications for understanding how family groups are formed, which may lend clarity to the evolution of cooperative breeding in this and other species.

## Materials and methods

### Field collections

Chestnut-crowned babblers have been studied at Fowlers Gap in New South Wales, Australia, a research station owned by the University of New South Wales, since 2004. Each year, breeding is monitored across the field site, all adults are captured, measured, and bled, and chicks are measured and bled one to three times before fledging. Although population size fluctuates by year, we monitor around 500 individuals per year, on average. For this study, samples were collected during the breeding seasons of 2014 and 2015. This study was conducted in accordance with *Animal Behaviour*’s guidelines for ethical treatment. The protocol was approved by Macquarie University’s Animal Care and Ethics Committee (license no. 06/40A), the NSW National Parks and Wildlife Service, and the Australian Bat and Bird Banding Scheme.

For this study, we used blood samples from individuals at hatching, just before fledging, and adults. Samples were chosen for individuals who had all necessary samples after adult phenotype (dispersive or philopatric) was known to ensure individuals were not related. All blood samples were stored in 95% ethanol at room temperature until DNA extraction. To sample chicks at hatching, eggs were removed from the nest during the latter half of the incubation period, replaced with model eggs, and artificially incubated in a rocking incubator (Brinsea Octogon 20) at 37.5°C and 42–45% humidity until hatching. Within an hour of hatching, chicks were weighed, had a blood sample taken, and toenails clipped for individual identity. Chicks were then returned to the nest and ringed at 10d, before the toenail clip grew out. A second blood sample and morphological measurements were taken at ~15d. After samples were collected, chicks were returned to the nest and allowed to fledge naturally; chestnut-crowned babbler chicks fledge between 18 and 21d. Finally, adults were captured with mistnets when morphological measurements and blood samples were collected.

### Molecular methods

DNA was extracted from preserved blood samples using Gentra PureGene DNA (Qiagen) extraction kit according to manufacturer’s instructions. Methylation was determined using an epiRADseq approach on an Ion Torrent Personal Genome Machine (Thermo Fisher Scientific) [36, 37]. epiRADseq is a reduced-representation library based approach to look at genome wide levels of methylation [37]. It uses a combination of next-generation sequencing technology with the methylation sensitive restriction enzyme *HpaII* (New England Biolabs) to measure DNA methylation by read count variation in resulting fragments. Significant differences in loci recovery indicate differences in methylation across individuals. Because the epiRADseq method is based on methylation-sensitive enzymes that do not cut the genome, and therefore do not produce a sequenced read, when a cut site is methylated, greater sequencing depth at a locus indicates lower methylation at that locus. Although genotypic variation in addition to differences in methylation can account for variation in loci sequencing, as genotypes are consistent within individual over time, any comparison looking at the same individual would not run into these problems. Although we acknowledge that other techniques exist to measure DNA methylation that are better suited for the identification of specific genes or sites of methylation (i.e. bisulfite sequencing based methods), we chose epiRADseq because, at the time of this study, no reference genome was available for chestnut-crowned babbler, thus epiRADseq maximized our efficiency to generate whole-genome levels of methylation to screen differences among and within individuals. After restriction digestion with *HpaII*, we ligated double stranded barcoded adaptors and y-adaptors of the Ion Torrent IonXpress sequences. We conducted emulsion PCR using the Ion PGM-Hi-Q-View OT2-200 kit following manufacturer’s directions on the Ion Express OneTouch2 platform. We then sequenced resulting fragments with the Ion PGM-Hi-Q-View Sequencing 200 Kit using an Ion 316v2 BC Chip following the manufacturer’s protocol.

We determined genome wide levels of methylation in 57 samples from 24 individuals. Of those, eight individuals (four philopatric individuals and four dispersive individuals) were sequenced at the three time points. Individuals were considered philopatric if they remained with their natal social group as adults and dispersive if they moved to become part of an unrelated group in their first year. All individuals originated from unrelated social groups of between three and 14 adults (mean ± SD = 6.5 ± 3.3 with no difference in social group size between dispersive and philopatric individuals: t = -0.5, p = 0.85). Further, birds used did not vary in mass at fledging (mean ± SD = 38.3 ± 1.1 (philopatric) and 37.2 ± 1.0 (dispersers); t = 2.08, p = 0.13).

### Data analysis

We demultiplexed epiRADseq runs and performed quality control (QC) using Torrent Suite version 4.4.3. We then trimmed adaptor sequences (retaining 50–150 bp) and performed a de novo assembly using Geneious v. 11.1.4, thus constructing a pseudo-reference sequence from the generated assembly. We mapped individual fragments with BWA [38, 39] and used featureCounts [40] to determine read counts of fragments for 50 bp bins. To generate the proportion of loci that were methylated at each time point for each individual, we collapsed read count data into a binary matrix to compare methylated loci (as zero; no fragments observed) with unmethylated loci (as one; at least one fragment observed). To determine how methylation status of each loci changed between ages within an individual (i.e. gained or lost methylation), we analyzed the difference in sequence reads at each fragment and determined how that changed through time.

We compared total proportion of methylated loci to age and dispersal status using a Bayesian generalized linear mixed model (GLMM) with a beta likelihood and a logit link (S1 File). Further, we determined how methylation state changed over time with a GLMM with a binomial likelihood and log link (S1 File). In all models, individual was a random effect to account for repeat sampling of individuals. Prior values for both models were chosen using prior predictive checks to ensure prior predictions generated reasonable upper and lower bounds [41] (S1 File and S1 Fig). Both models were fitted using the *brms* package [42] via Hamiltonian Monte Carlo in *rstan* [43]. We ran four chains of 2000 iterations each. The first 1000 iterations of each chain were discarded as warmup. We used posterior predictive checks to test model performance [41] and checked chains for convergence by ensuring r-hats were less than 1.1. Finally, using the larger dataset (n = 24; 11 females, 13 males), we found no difference in the proportion of genome methylation between males and females (S2 Fig) and thus sex was not included in the models.

We determined how methylation state changed over time similarly using a GLMM with a binomial likelihood and log link. In this model, the response variable was the number of loci that changed methylation status (gained or lost methylation) out of the total number of loci examined. The predictor variables were dispersal status, age transition (i.e. from hatchling to fledgling or from fledgling to adult), and their interaction with individual as a random effect. Priors were *N*(0, 1) for the intercept, *N*(0, 1) for the betas, and Cauchy(0, 1) for the standard deviation.

As prior specifications become increasingly important in models with small samples sizes, as those used here, we conducted a prior sensitivity analysis [44] to ensure we were not over-generalizing our results from a small sample size (S1 File, S3 and S4 Figs). All data and code are available at gttps://github.com/jswesner/liebl_birds.

## Results

Following QC and trimming, the sequence reads formed a pseudo-reference sequence of 21,335,109 bases. Mapping to the pseudo-reference sequence resulted in 10,138 to 27,817 unique mapped reads per individual (i.e. loci). After binning, we removed all loci with a sequenced fragment in less than 10% of screened individuals generating 40,361 bins, which were treated as loci and were used for subsequent analysis.

### Genome wide levels of methylation

**Fig 1:**
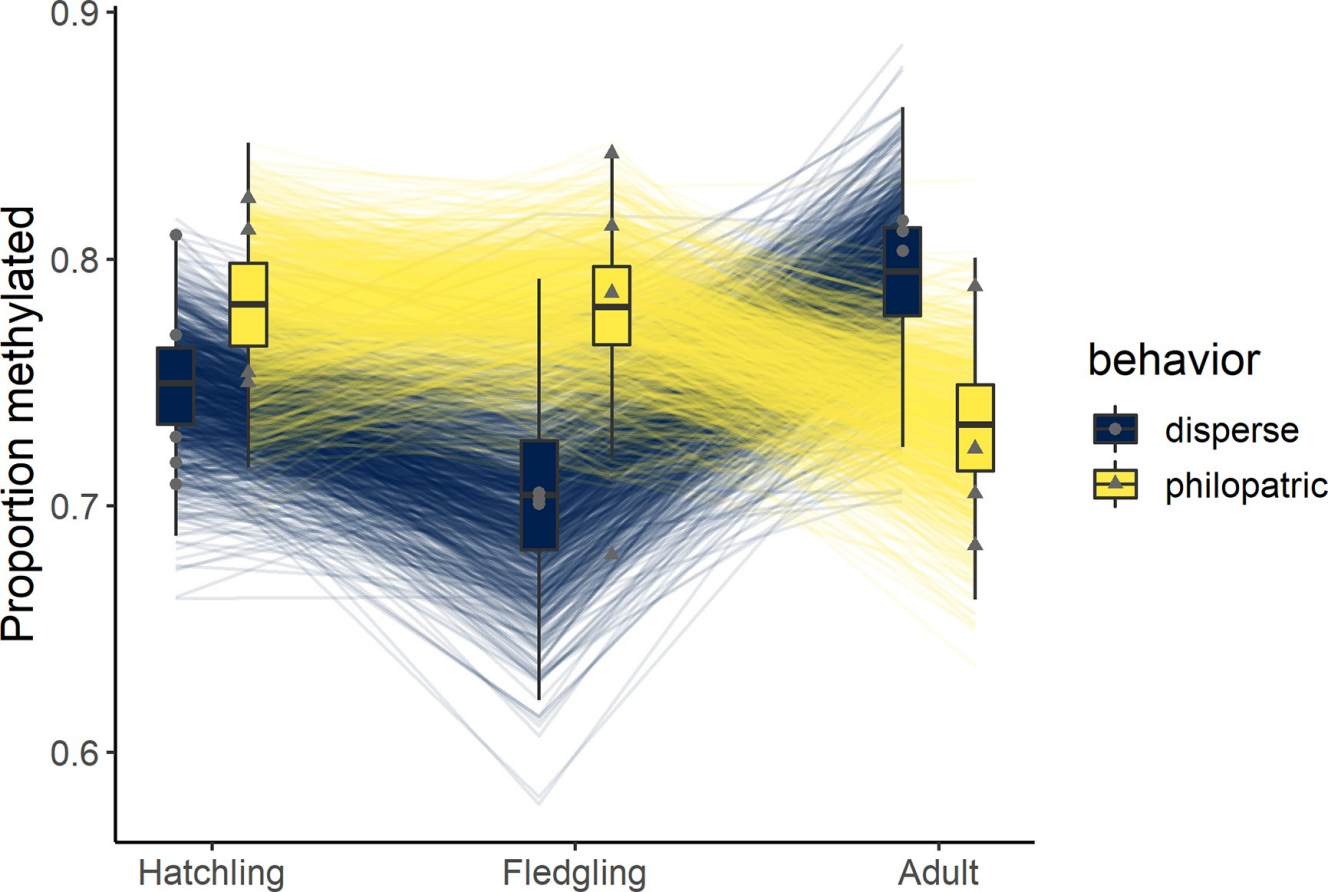
Mean proportion of methylated loci at hatching, fledging, and 1 year for dispersing and philopatric chestnut-crowned babblers. Boxplots represent summaries of the posterior distribution, lines connect estimated values in each stage across 1000 iterations of the posterior distribution, and data points are the raw data.

At hatching, individuals that ultimately dispersed had similar proportions of genome-wide methylation as individuals that remained with their natal group (mean ± SD of the posterior distribution = 0.75 ± 0.02 and 0.77 ± 0.03 respectively; Fig 1; Table 1). In contrast, at fledging, dispersers had a lower average proportion of methylated loci (0.7 ± 0.03 compared to 0.78 ± 0.02 methylated loci in philopatric individuals; Fig 1; Table 1). This corresponded to a mean difference of 0.08 ± 0.04 (95% CrI: 0.01 to -0.15) with a >96% probability that dispersive individuals had lower genome wide methylation than philopatric individuals at fledging. By the adult stage, the difference between philopatric and dispersive individuals had reversed (0.8 ± 0.02 in dispersive individuals versus 0.74 ± 0.03 for philopatric individuals; Fig 1; Table 1), with a 96% probability that dispersive individuals had higher genome-wide methylation than philopatric individuals as adults.

**Table 1:** Summaries proportion of methylated loci for hatchlings (H), fledglings (F), and adults (A) that either dispersed (D) or remained philopatric (P). Values represent the mean, standard deviation (sd) and upper and lower 95% credible intervals from a Bayesian GLMM.

age	dispersed	mean	sd	low95	high95
H	D	0.75	0.02	0.71	0.79
H	P	0.77	0.03	0.72	0.82
F	D	0.71	0.03	0.65	0.76
F	P	0.81	0.02	0.76	0.85
A	D	0.8	0.02	0.75	0.84
A	P	0.74	0.03	0.69	0.8

### Changes in methylation over time

**Fig 2:**
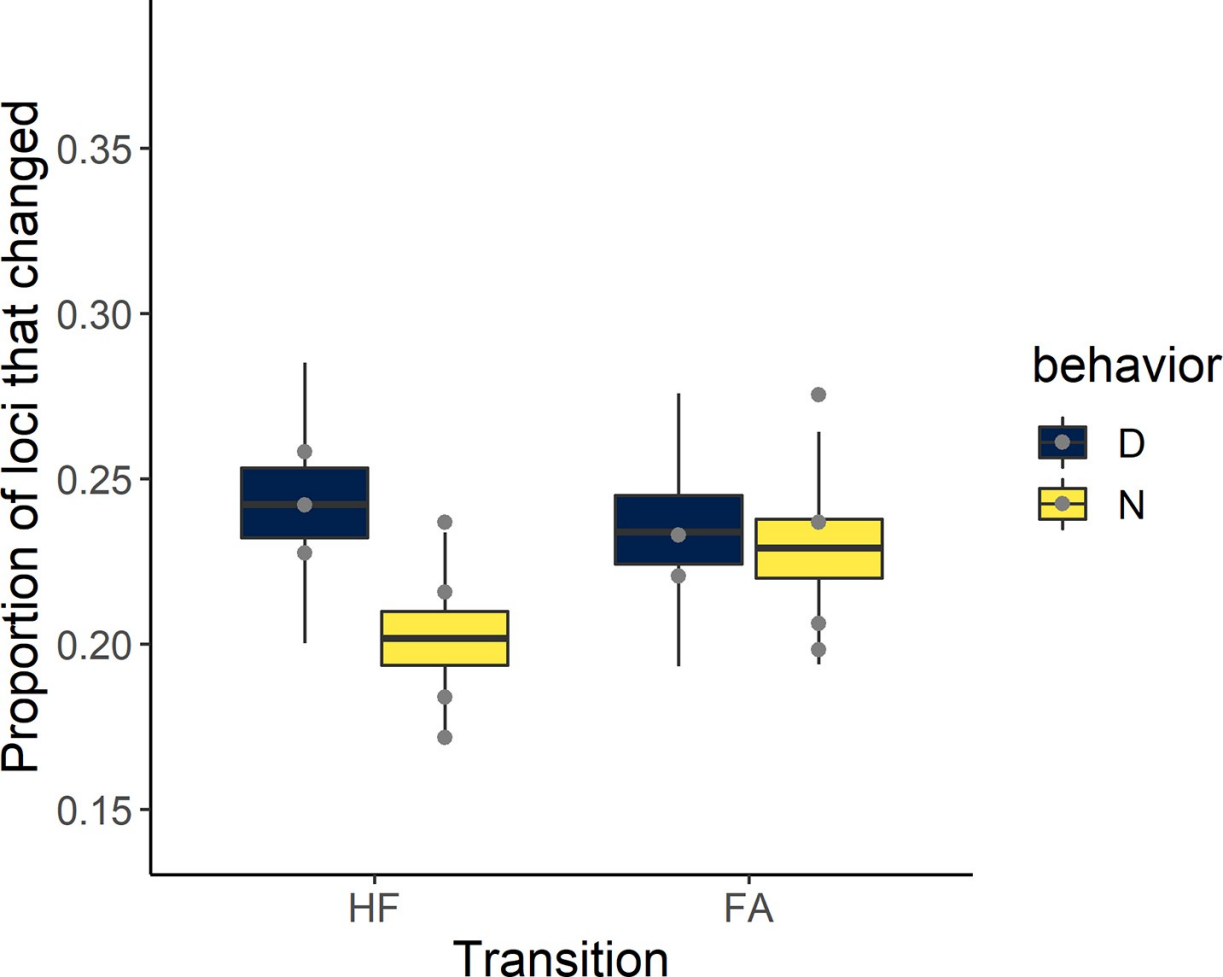
Mean proportion of loci that changed methylation status (gained or lost methylation) during the transition from hatchling to fledgling or from fledgling to adult. Boxplots represent summaries of the posterior distribution for dispersing and philopatric birds and data points are the raw data.

Although most (78%, on average) loci did not gain or lose methylation between sampling (i.e. between hatching and fledging or between fledging and adult), dispersers had a greater number of loci that either changed their methylation by either gaining or losing methylation than philopatric birds, particularly between hatching and fledging (Table 2). Specifically, 24 ± 2% of the loci in dispersers either gained or lost methylation between hatching and fledging, whereas only 20 ± 1% of loci changed in philopatric birds (Fig 2); the probability that this difference was greater than zero was >96%. Interestingly, of the loci that changed between hatching and fledging, 897 loci gained methylation and 375 loci lost methylation across all dispersive individuals, whereas only 79 loci gained and 38 loci lost methylation across all philopatric individuals. Between fledging and 1 year, changes in methylation were more comparable between dispersers and philopatric individuals (24 ± 2% and 23 ± 2% respectively), with only a 60% probability that this difference was greater than zero.

**Table 2:** Summaries of the proportion of loci that changed methylation status in birds that dispersed (D) or remained philopatric (P) during transitions between the hatchling-fledgling (HF) or fledgling-adult (FA) stage. Values represent the mean, standard deviation (sd) and upper and lower 95% credible intervals from a Bayesian GLMM.

Transition	Behavior	mean	sd	low95	high95
HF	D	0.24	0.02	0.28	0.21
HF	P	0.20	0.01	0.23	0.18
FA	D	0.24	0.02	0.28	0.20
FA	P	0.23	0.02	0.26	0.20

## Discussion

Epigenetic changes during development are influential in how phenotypes are expressed throughout life. One example, in eusocial insects, has shown that the propensity to cooperate or breed is driven by methylation derived during development [18]. In another cooperative breeder, superb starlings (*Lamprotornis superbus*), differential methylation driven by differences in developmental environment influenced adult fitness levels in males [24]. However, whether changes in methylation influence delayed dispersal decisions, and by extension, cooperation, is yet unknown. Reduced methylation of the genome, allowing greater flexibility of gene expression, can increase phenotypic plasticity of certain traits [15, 19, 45]; this may be particularly beneficial for individuals with an unpredictable future environment, such as those expected for dispersing individuals. On the other hand, as social, and often physical, environments do not change between fledging and adulthood in birds that remain philopatric, those individuals that remain with their natal group may not benefit as much from increased plasticity as those dispersing into an unknown environment. We show that genome-wide levels of methylation in the blood were lower in offspring that dispersed than those that remained philopatric in their first year. Despite no difference at hatching, methylation status changed at a greater number of loci in dispersers between hatching and fledging resulting in lower levels of overall methylation just before fledging. Further, the number of loci that either gained or lost methylation across all dispersers, but in not philopatric individuals, was higher than those found in philopatric but not dispersive individuals. Thus, the epigenetic changes derived during development may prepare offspring for their future environments- whether they be predictable for philopatric individuals or unpredictable for dispersive individuals. Although we cannot yet determine the specific environmental cues generating these differences, given the amount of change seen during the post-hatching, pre-fledging period, the results suggest this developmental window may be particularly important for individuals in future dispersal decisions.

Research in non-cooperatively breeding species suggests a relationship between dispersal and particular gene types such as lipid metabolism and antigen defense [5]. It is likely that some of the differentially methylated loci identified here may also be related to dispersal. However, the epiRADseq approach used does not quantify differential methylation at particular genes; rather, it measures broad differences in methylation patterns across the whole genome, and has been used to measure general, ecological epigenetic patterns across a range of systems [37, 46, 47]. Despite the clear differences we report between dispersive and philopatric individuals in the genome-wide levels of methylation at fledging, we are unable to elucidate whether and which of the differentially methylated loci are found in functionally relevant coding regions of the genome. Future work should use more targeted approaches such as bisulfite sequencing to test for more specific, gene-level differences in methylation. Such studies are still rare in ecological research, but they are important to identify the specific mechanisms by which methylation can influence phenotypes. Additionally, although the epigenetic patterns derived using blood samples (particularly those primarily from nucleated red blood cells as would be expected from the avian samples collected here) might not be representative of all tissues [48–50], some studies have shown correlations between methylation patterns in the blood and other tissues [51, 52]. Further, using peripheral samples has successfully revealed links between epigenetic variation and behavior in other species (primarily humans) [53–56]. Using these peripheral samples has been shown to be informative in describing the link between the environment and the genome and, perhaps more importantly, allows repeat sampling of the same individual over time to analyze how environments are related to *changes* in methylation and what the subsequent influence on phenotype is.

Dispersal can be influenced by a myriad of factors relating to individual internal and external environment including the genome [1–4], hormonal state [57, 58], personality [59], and condition [60–62]. Many environmental cues experienced during development influence behavioral phenotypes throughout life (reviewed in [63]), including dispersal [3, 64–66]; importantly, many of these phenotypes are derived via epigenetic changes to the genome. Notably, cooperative breeding is more prevalent in species living in harsh and unpredictable environments [6]; further, harsh environments may be expected to play an outsized role in shaping environmentally influenced phenotypes. Although we show no evidence of sex differences in methylation patterns between males and females here, some evidence exists that developmental environment can differentially influence methylation dependent on sex [24]. As with most avian cooperative systems, female offspring tend to be more dispersive whereas males tend to be more philopatric [8, 34]. Here, although dispersive individuals were split between males and females, the philopatric group included only males. Future work might address the interaction between sex and developmental environment on adult behavior.

One particularly important environmental cue experienced during development is parental care [27, 67]; the amount and quality of parental care can vary both within (with parents additional care dependent on sex, age, size, or other offspring attributes [68, 69]) and among broods. Variation in parental care is especially high in cooperatively breeding species like chestnut-crowned babblers, where offspring are raised by a variable number of adults [70]. Although the links between variable parental care and methylation patterns and adult phenotype is unknown, given the amount of variation in parental care demonstrated across offspring, particularly in cooperatively breeding societies, it is possible that it plays an important role in developmentally-determined phenotypes. However, in addition to parental care during the nestling period, many other developmental environments are experienced by chicks, including (but not limited to) territorial quality, the post-fledging social environment, environmental stability, and climatatic variables during dispersal; all of which may also contribute to dispersal decisions.

If differential methylation is indeed driven by variation in parental care in chestnut-crowned babblers, this could reveal a molecular mechanism by which parents and helpers could adaptively and plastically adjust offspring phenotypes to best match the needs of the offspring as well as the group. Given the numerous, and often conflicting cues individuals experiences aduring development, predicting offspring disperal is not only a challenge for scientists, but also for the animals themselves. Using molecular mechanisms such as epigenetic processes that interact with both the environment and genome may help guide adaptive decions regarding whether or not to disperse.

## Conclusions

Ecological epigenetics is a relatively new field of study and thus many of the techniques long available to medical researchers are just becoming accessible to molecular ecologists. Despite this, these results fit into a growing body of evidence indicating that differences in epigenetic markers can lead to predictable phenotypic variation across non-model organisms. Although we do not yet know what specific genes are being epigenetically controlled here, or what specific environmental cues are driving molecular differences between dispersive and philopatric individuals, this research paints a picture showing that epigenetic marks, particularly those derived during nestling development, may be important in determining dispersal decisions in chestnut-crowned babblers. Future research should focus on identifying the specific environmental cues that trigger variation in methylation as well as the specific genes that are differentially methylated between dispersive and philopatric individuals. Because dispersal decisions can alter the fitness landscape of cooperatively breeding species, understanding the molecular mechanisms that drive dispersal, and how those mechanisms are derived may shed light on how such a seemingly paradoxical behavior has evolved.

## Supporting information

S1 Fig Prior and posterior comparisons. The top panels show the model results before including the data (i.e. generated only from the prior distribution). The bottom panels show the same models, but now including the data (i.e. generated from the posterior distribution. Circles are means and error bars are 95% CrI.
(TIF)

S2 Fig Proportion of methylated loci in male and female birds. Boxplots summarize the posterior distribution from a Beta GLMM, and dots are the raw data.
(TIF)

S3 Fig Parameter estimates from a prior sensitivity analysis. The model presented in the main text has a prior standard deviation of 1, *N*(0,1), and is shown with the large green circle. The other models contain parameter estimates after adjusting the prior and re-running the model. Values less than 1 are more restrictive priors. Values greater than 1 are less restrictive priors compared to the prior for the main model. Values for the yellow triangle represent parameter estimates from maximum likelihood using the *lme4* package. These results are roughly akin to running a Bayesian model with a standard deviation of infinity–Inf (Max Lik) on all priors.
(TIF)

S4 Fig Conditional effects from a prior sensitivity analysis. The model presented in the main text—1 (MS)—has a prior standard deviation of 1 (*N*(0,1)). The other models show alternative results after adjusting the prior and re-running the model. Values less than 1 are more restrictive priors. Values greater than 1 are less restrictive priors compared to the prior for the main model. Results from maximum likelihood use the *lme4* package–Inf (Max Lik). Maximum likelihood results are roughly akin to running a Bayesian model with a standard deviation of infinity on all priors.
(TIF)

S1 File Supplemental methods. This file includes descriptions of Bayesian model analysis, sex differences in methylation, and the power of the Bayesian statistics for this analysis compared to a frequentist approach.
(DOCX)
