# Supplementary figures and images for: Methylation patterns at fledging predict delayed dispersal in a cooperatively breeding bird
Source: PLoS One. 2021 Jun 4;16(6):e0252227. doi: 10.1371/journal.pone.0252227 (PMC8177507; doi:10.1371/journal.pone.0252227)

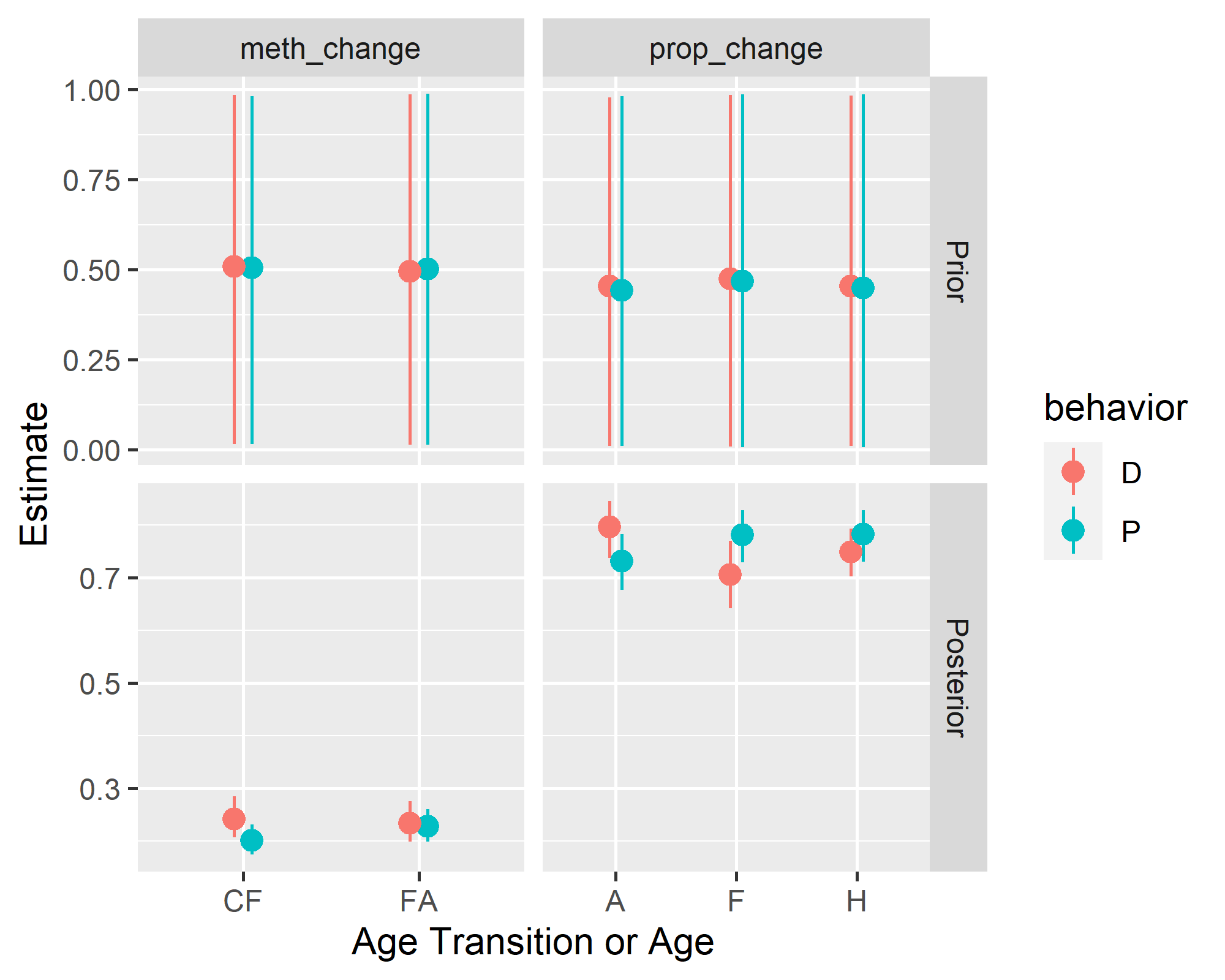

Supplement: S1 Fig — The top panels show the model results before including the data (i.e. generated only from the prior distribution). The bottom panels show the same models, but now including the data (i.e. generated from the posterior distribution. Circles are means and error bars are 95% CrI. (TIF) [file pone.0252227.s001.tif]

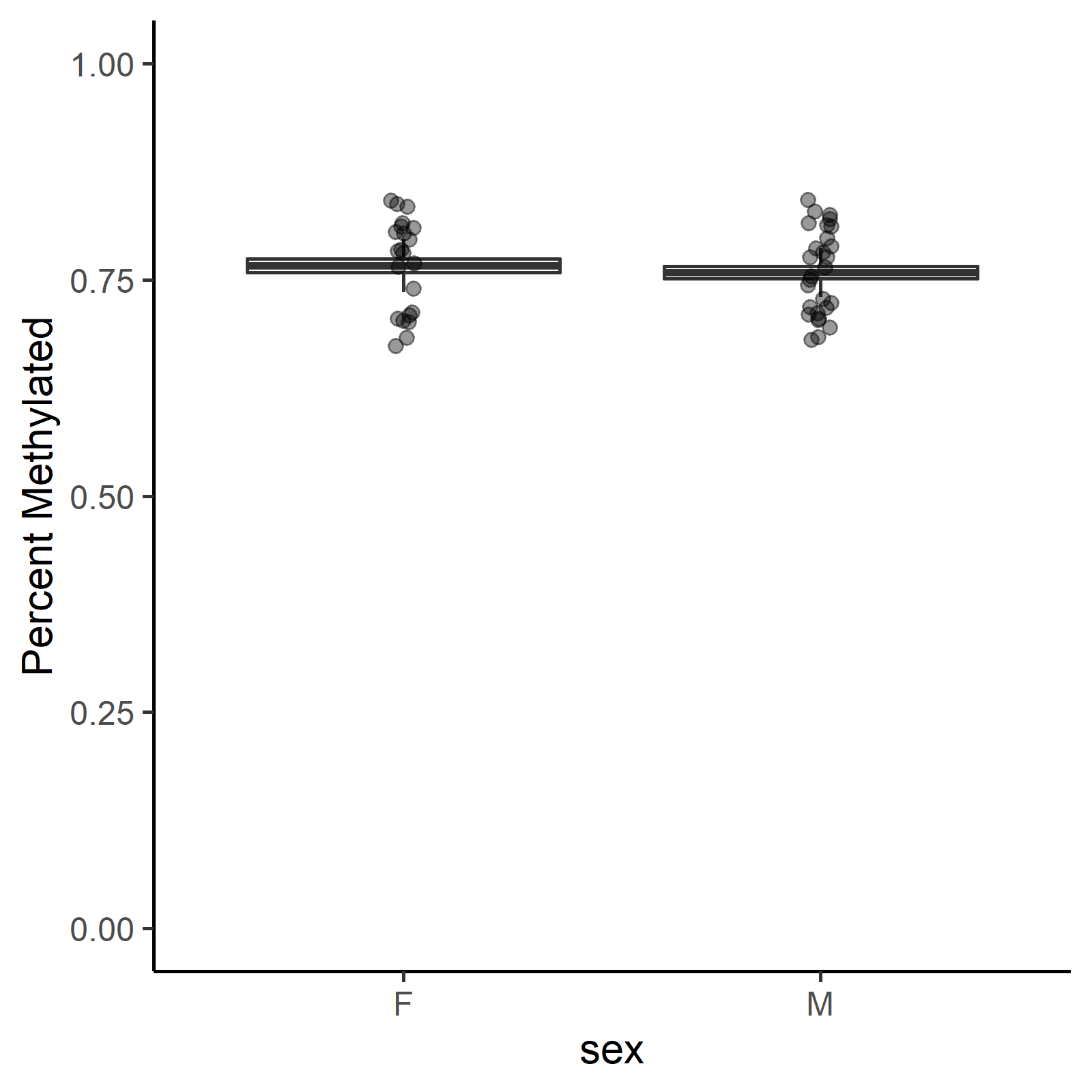

Supplement: S2 Fig — Boxplots summarize the posterior distribution from a Beta GLMM, and dots are the raw data. (TIF) [file pone.0252227.s002.tif]

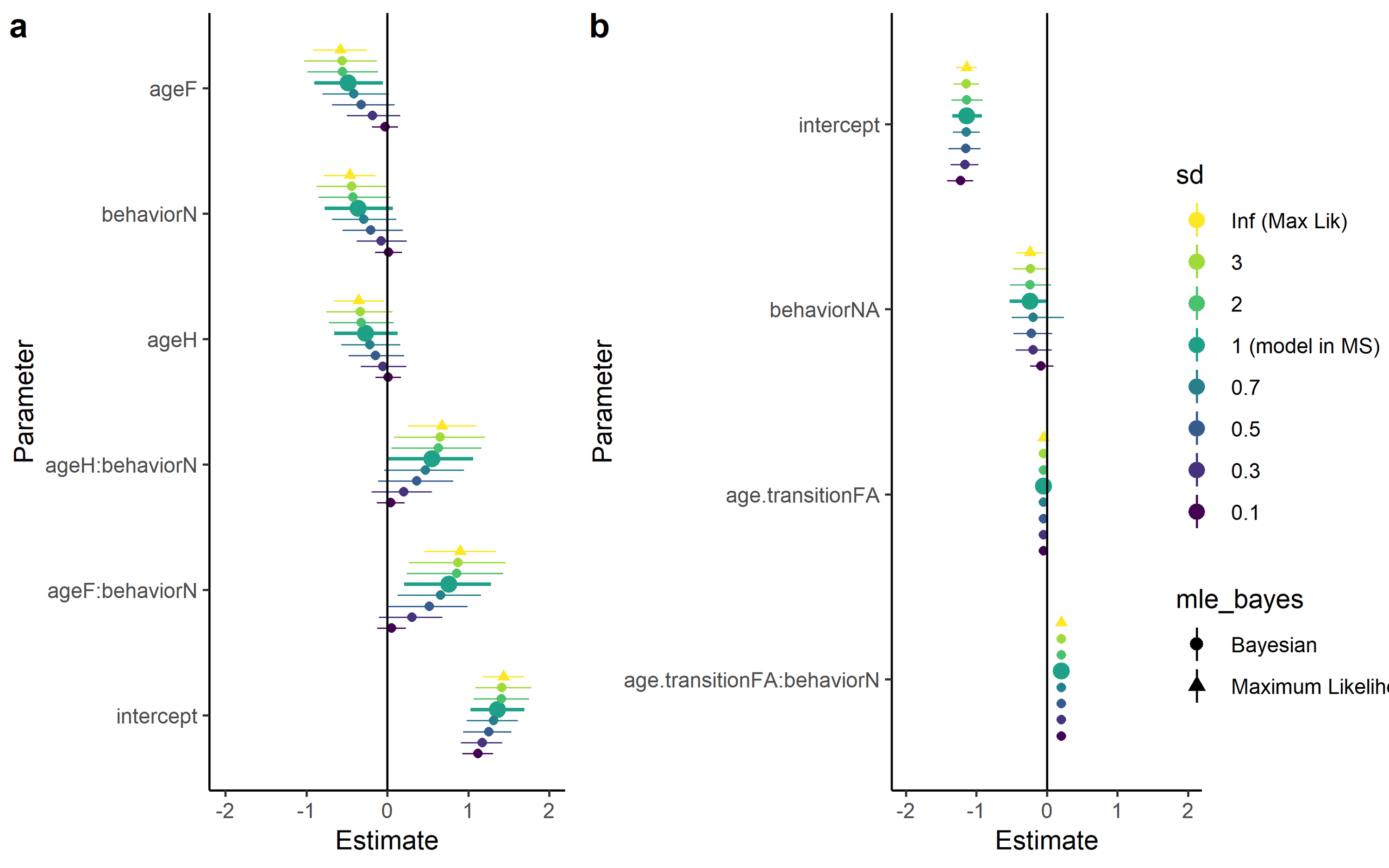

Supplement: S3 Fig — The model presented in the main text has a prior standard deviation of 1, N(0,1), and is shown with the large green circle. The other models contain parameter estimates after adjusting the prior and re-running the model. Values less than 1 are more restrictive priors. Values greater than 1 are less restrictive priors compared to the prior for the main model. Values for the yellow triangle represent parameter estimates from maximum likelihood using the lme4 package. These results are roughly akin to running a Bayesian model with a standard deviation of infinity–Inf (Max Lik) on all priors. (TIF) [file pone.0252227.s003.tif]

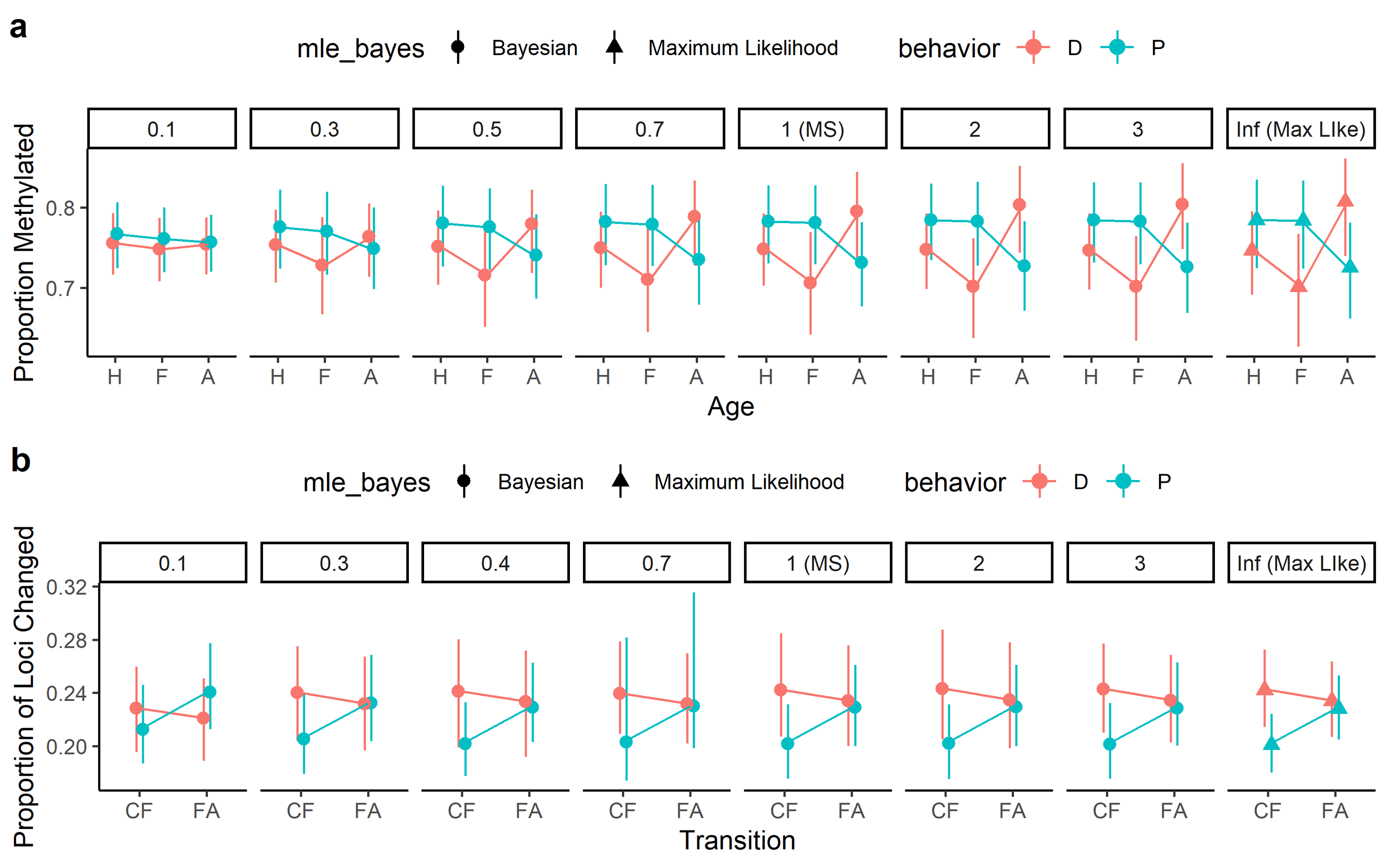

Supplement: S4 Fig — The model presented in the main text—1 (MS)—has a prior standard deviation of 1 (N(0,1)). The other models show alternative results after adjusting the prior and re-running the model. Values less than 1 are more restrictive priors. Values greater than 1 are less restrictive priors compared to the prior for the main model. Results from maximum likelihood use the lme4 package–Inf (Max Lik). Maximum likelihood results are roughly akin to running a Bayesian model with a standard deviation of infinity on all priors. (TIF) [file pone.0252227.s004.tif]
