# Supplementary material for: Methylation patterns at fledging predict delayed dispersal in a cooperatively breeding bird
Source: PLoS One. 2021 Jun 4;16(6):e0252227. doi: 10.1371/journal.pone.0252227 (PMC8177507; doi:10.1371/journal.pone.0252227)
Supplement: S1 File — This file includes descriptions of Bayesian model analysis, sex differences in methylation, and the power of the Bayesian statistics for this analysis compared to a frequentist approach. (DOCX) [file pone.0252227.s005.docx]

**S1 File**

**Statistical Models**

We compared total proportion of methylated loci to age and dispersal status using a Bayesian generalized linear mixed model (GLMM) with a beta likelihood with a logit link. The response variable was the proportion of methylated loci, the predictor variables were age, dispersal status, and their interaction; individual identity was fitted as a random intercept. Priors were *N*(0, 2) for the intercept, *N*(0, 1) for the betas, HalfCauchy(0, 1) for the standard deviation, and gamma(0.01, 0.01) for phi. The model structure and priors were:

*P_i_* ~ Beta(*mu_i_, phi*)

logit(*mu_i_*) = *α* + *α*_Ind[i]_ + *β_A_A_i_* + *β_D_D_i_* + *β_AD_A_i_D_i_*

*α* ~ Normal(0, 2)

*α*_Ind[i]_ ~ Normal(0, *σ*_Ind_)

*β_X_* ~ Normal(0, 1)

*σ*_Ind_ ~ HalfCauchy(0, 1)

*phi* ~ Gamma(0.01, 0.01)

*P_i_* is the proportion of methylated loci in the *i*th individual, *mu_i_* is the mean parameterized as shape1/(shape1 + phi) from the parameters of the beta distribution, *α* is the intercept, *α*_Ind[i]_ is the random effect (varying intercept), *β*’s are the coefficients for age (*A*) or dispersal (*D*), *σ*_Ind_ is the prior for the sd of the random effect, and *phi* is the second parameter of the beta distribution.

The second analysis, determining how methylation state changed over time, was completed with a GLMM with a binomial likelihood and logit link. In this model, the response variable was the number of loci that changed methylation status (gained or lost methylation) out of the total number of loci examined. The predictor variables were dispersal status, age transition (i.e. from hatchling to fledgling or from fledgling to adult), and their interaction. Priors were *N*(0, 1) for the intercept, *N*(0, 1) for the betas, and Cauchy(0, 1) for the standard deviation. The model structure was

*C_i_* ~ Binomial(*n_i_, p_i_*)

logit(*p_i_*) = *α* + *α*_Ind[i]_ + *β_At_At_i_* + *β_D_D_i_* + *β_AtD_At_i_D_i_*

*α* ~ Normal(0, 1)

*α*_Ind[i]_ ~ Normal(0, *σ*_Ind_)

*β_X_* ~ Normal(0, 1)

*σ*_Ind_ ~ HalfCauchy(0, 1)

*C_i_* is the number of loci that changed methylation status out of the total number of loci examined (*n_i_*) for the *i*th individual, *p_i_* is the probability of changing methylation status, *A_t_* is the age of transition with two levels (hatchling to fledgling or fledgling to adult), and all other parameters are the same as the previous model.

**Prior Predictive Distribution**

To determine whether our prior choices were reasonable before seeing the data, we plotted the outcomes of the model using the prior predictive distribution. This represents the model outcome when fit using only simulations from the prior, not the data (Gabry et al. 2019). The result is shown in S1 Fig. Prior to seeing the data, the model expects a wide possibility of outcomes ranging from ~ 0 to 100% methylation change or 0 to 100% change in the proportion of loci that are methylated. In addition, there is no expectation of difference among treatments *a priori*, as indicated by the similarity of prior predictions across the treatment groups.

After seeing the data, there is a clear change from the prior to the posterior. The difference in the prior and posterior predictions is indicative of the amount of information learned from the data. In this case, the posterior distributions are far less uncertain than the prior distributions, even with a relatively small sample size.

**Differences in methylation between sexes**

To determine whether males and females contained different levels of methylation, we compared the percent of loci that were methylated using a Bayesian GLMM with a Beta likelihood and logit link. The response variable was the proportion of loci methylated and the predictor variable was sex with a random intercept of individual. S2 Fig shows the result, indicating no difference in methylation between sexes, with each having ~76% of loci methylated.

**Prior Sensitivity Analysis**

*Approach*

Prior specifications become increasingly important in models with small sample sizes. Therefore, to determine how sensitive our results were the priors, we conducted a prior sensitivity analysis (Hobbs and Hooten 2015). To do this, we re-ran both models (see text for model specifications) six times, each time changing the prior standard deviation for the *β* parameters to make them either more informative (smaller value of the prior sd) or less informative (larger value of the prior sd) (Hobbs and Hooten 2015, Korner-Nievergelt et al. 2015) relative to the original prior of *N*(0,1). In addition, we ran each model under a maximum likelihood framework using the *lme4* (Bates et al. 2014) or *glmmTMB* (Brooks et al. 2017) packages for the binomial model or beta model, respectively. Maximum likelihood estimates are roughly akin to Bayesian estimates with a uniform prior between -∞ and +∞ (McElreath 2020).

To compare the results, we plotted the estimates of individual parameters from each model separately (S3a,b Fig). In addition, we plotted the conditional effects from each model (S4a,b Fig). The conditional effects are the means and 95% confidence/credible intervals of the predictions we were interested in. These plots replicate the same plots as Figs 1 and 2 in the main text but iterate over different priors to show how the results change in response to prior specification.

*Results*

For the first model (Proportion of loci methylated at each age and dispersal status), the parameter estimates converged towards zero as the prior became more informative (smaller sd) (S3a Fig). However, the qualitative result in the main model was robust to all but the two most restrictive priors (S4a Fig). That is, our qualitative conclusions about the proportion of methylated loci differing between dispersed and philopatric fledglings would not have changed, as indicated by the non-overlap of the 95% intervals with the mean of either group when the prior standard deviation is 0.5 or higher.

For the second model (Proportion of loci that changed methylation status), the parameter estimates are less sensitive to prior specifications (S3b Fig). As shown in S4b Fig, the parameter estimates do not appreciably change until the prior sd is at its most restrictive setting (0.1). Similarly, the qualitative result relative to the main model is similar across all parameter settings except for *N*(0, 0.1).

As expected, regardless of prior specification, all estimates from the Bayesian models are more conservative than the maximum likelihood estimates. This is due to the priors being centered on zero. As a result, the prior expectation is always weighted towards a conservative assumption of no difference in methylation or no change in methylation. Whether that prior has a meaningful influence on the posterior depends on how restrictive the standard deviation is. By contrast, the maximum likelihood estimate is derived entirely from the data and has no inherent pull towards zero (i.e. it has an implicit uniform prior). As a result, any risks of over-generalizing from a small sample size are highest under the maximum likelihood scenario.

**References - Supplementary**

Bates, D., Mächler, M., Bolker, B., & Walker, S. (2014). Fitting linear mixed-effects models using lme4. *arXiv preprint arXiv:1406.5823*.

Brooks, M. E., Kristensen, K., van Benthem, K. J., Magnusson, A., Berg, C. W., Nielsen, A., ... & Bolker, B. M. (2017). glmmTMB balances speed and flexibility among packages for zero-inflated generalized linear mixed modeling. *The R journal*, *9*(2), 378-400.

Gabry, J., Simpson, D., Vehtari, A., Betancourt, M., & Gelman, A. (2019). Visualization in Bayesian workflow. *Journal of the Royal Statistical Society: Series A (Statistics in Society)*, *182*(2), 389-402.

Gelman, A., Carlin, J. B., Stern, H. S., Dunson, D. B., Vehtari, A., & Rubin, D. B. (2013). *Bayesian data analysis*. CRC press.

Hobbs, N. T., & Hooten, M. B. (2015). *Bayesian models: a statistical primer for ecologists*. Princeton University Press.

Korner-Nievergelt, F., Roth, T., Von Felten, S., Guélat, J., Almasi, B., & Korner-Nievergelt, P. (2015). *Bayesian data analysis in ecology using linear models with R, BUGS, and Stan*. Academic Press.

McElreath, R. (2020). *Statistical rethinking: A Bayesian course with examples in R and Stan*. CRC press.
